# Author Response to Peer Reviews of “A Physical Activity Mobile Game for Hematopoietic Stem Cell Transplant Patients: App Design, Development, and Evaluation”

**DOI:** 10.2196/28334

**Published:** 2021-04-13

**Authors:** Shannon Cerbas, Arpad Kelemen, Yulan Liang, Cecilia Sik-Lanyi, Barbara Van de Castle

**Affiliations:** 1 The Sidney Kimmel Comprehensive Cancer Center Johns Hopkins University Baltimore, MD United States; 2 University of Maryland Baltimore, MD United States; 3 University of Pannonia Veszprém Hungary

**Keywords:** cancer, mobile app, gamification, bone marrow transplant, alpha testing, physical activity

*This is the authors’ response to peer-review reports for "A Physical Activity Mobile Game for Hematopoietic Stem Cell Transplant Patients: App Design, Development, and Evaluation".*

## Response to Round 1 Reviews

The authors of the manuscript [1] are grateful to the editor and reviewers [2,3] for their invaluable input and feedback.

### Response to Reviewer P [2]

#### Specific Comments

##### Major Comments

###### Introduction

1. Agreed, this was changed.
2. Agreed, this was changed.
3. This was added.
4. This population varies between inpatients and outpatients so an app for this type of varied use is appropriate. A discussion was added.
5. We agree. New discussion points were added.
6. Yes, continued game play requires walking a clinically set number of steps. We further explained this in the updated manuscript.

###### Methods

1. We changed the text from “user-centered design” to “we focused on the intended users.” We achieved this by (1) collecting data from the intended users about games they already enjoy, and we chose Candy Crush as a template for our game design; (2) a survey of clinical nurses who worked with the target patient population to tailor the game to the intended users; (3) evaluation of the game and step counter by nurses, some of whom worked with the target population; and (4) the project is scheduled to be played by the intended users to collect data from them for future changes.
2. We added discussion points on this.
3. Hierarchical cluster analysis and exploratory factor analysis are quantitative analysis methods. We added more details about this in the *Methods* section. The results and interpretations are included in the *Results* section.

###### Results

1. We added more information about the characteristics of the study sample.
2. There are no known normative values.
3. We moved this to the *Methods* section.

###### Discussion

1. While this would be an additional important topic to cover, the review we received from the journal states that this paper is already too long and we need to reduce the word count, which we agree with. It would be a lot of new content to add, with only marginal relevance and little benefit for this paper. Therefore, we prefer not to add other studies to the *Discussion* section at this time. We discussed some other related studies in the *Introduction* section.

### Response to Reviewer V [3]

#### General Comments

1. We added more details about this.
2. We added more about the planned patient testing. Impact on walking behavior is a long-term goal. The past work that we report on in this paper is heavy on expert testing.
3. Thank you for pointing this out. The decision to release the game to the public is not a current consideration of this project. We are far from that decision. We need to administer it to our patients first, then analyze that data. We replaced the release part with the walking behavior test part since that should happen first.
4. We added text on the issues raised. Since the paper is already too long, maybe adding another figure is not needed.

#### Specific Comments

##### Abstract

1. We have made the changes accordingly.
2. We changed the paper to make this clear.

##### Introduction

1. Added.
2. We made the changes accordingly.
3. Age and Android smartphone ownership were added. We are targeting all of these patients in the clinical setting.

##### Methods

1. Added.
2. We made the changes accordingly.
3. Emails were removed.
4. Thanks for asking this. Yes, goal setting on walking is done by clinicians. We coded that into the frequency and number of steps needed to play the game. Self-monitoring and feedback are done through the online database we discussed. We have now added these aspects to the paper to clarify.
5. The purpose and the software used to conduct analyses were added in the *Methods* section. We made some changes and additions to the paper.
6. Thank you for pointing this out. We made these changes.

##### Discussion

1 and 2. Yes, these are all good points. We responded to each of these comments in the responses above and in the paper.

## Response to Round 2 Reviews

### Reviewer P [2]

1. All references to hypotheses were removed. However, note that testing those hypotheses are long-term objectives of this project that reach well beyond the scope of this paper and beyond our current programming and evaluation objectives.
2. Changes were made as suggested.
3. Discussion of this limitation was added as requested.
4. It was revised to remove ambiguity. The potential opportunities for improvement are already discussed in the paper. These include improvements to each of the 40 items surveyed in the expert usability evaluation; improvement on the step counter’s accuracy, robustness, cheat proofing, the game’s speed, movement of tiles, graphical appearance, “pause” and “back” buttons; the addition of a tutorial and other features; ease of use; optimizing for phone battery drain; developing our own open-source step counter; developing a separate step counter for iPhones and making the game compatible with iPhones; coding to exploit different hardware technologies; designing and developing artificial intelligence and machine learning algorithms to improve the step counter; training and fitting the step counter to individual users; usability improvements; bug fixes; user-driven modifications; changing the frequency and amount of steps for individual users; optional release of scores for public view, competitions among users, social community building; adjusting the game per HSCT patients’ feedback and recommendations; extending the project to a rigorous evaluation that includes feasibility, acceptability, patient walking behavior, and comparison of physical activity between Walking Warrior users and nonusers; and measuring impact on walking. These are all the major improvements our team can think of at this time.
5. Evidence was added.
6. Done.
7. Please see the newly added explanation and commentary in the *Results* section regarding the score differences. We also discussed this limitation in the *Discussion* section.
8. We made some changes and added more explanations.
9. Done.
